# What factors are associated with recent intimate partner violence? findings from the WHO multi-country study on women's health and domestic violence

**DOI:** 10.1186/1471-2458-11-109

**Published:** 2011-02-16

**Authors:** Tanya Abramsky, Charlotte H Watts, Claudia Garcia-Moreno, Karen Devries, Ligia Kiss, Mary Ellsberg, Henrica AFM Jansen, Lori Heise

**Affiliations:** 1 London School of Hygiene and Tropical Medicine, 15-17 Tavistock Place, London WC1H 9SH, UK; 2 WHO, Avenue Appia 20, 1211 Geneva 27, Switzerland; 3 International Centre for Research on Women, 1120 20thSt NW, Suite 500 North, Washington D.C. 20036, USA; 4 Geneva, Switzerland

## Abstract

**Background:**

Intimate partner violence (IPV) against women is a global public health and human rights concern. Despite a growing body of research into risk factors for IPV, methodological differences limit the extent to which comparisons can be made between studies. We used data from ten countries included in the WHO Multi-country Study on Women's Health and Domestic Violence to identify factors that are consistently associated with abuse across sites, in order to inform the design of IPV prevention programs.

**Methods:**

Standardised population-based household surveys were done between 2000 and 2003. One woman aged 15-49 years was randomly selected from each sampled household. Those who had ever had a male partner were asked about their experiences of physically and sexually violent acts. We performed multivariate logistic regression to identify predictors of physical and/or sexual partner violence within the past 12 months.

**Results:**

Despite wide variations in the prevalence of IPV, many factors affected IPV risk similarly across sites. Secondary education, high SES, and formal marriage offered protection, while alcohol abuse, cohabitation, young age, attitudes supportive of wife beating, having outside sexual partners, experiencing childhood abuse, growing up with domestic violence, and experiencing or perpetrating other forms of violence in adulthood, increased the risk of IPV. The strength of the association was greatest when both the woman and her partner had the risk factor.

**Conclusions:**

IPV prevention programs should increase focus on transforming gender norms and attitudes, addressing childhood abuse, and reducing harmful drinking. Development initiatives to improve access to education for girls and boys may also have an important role in violence prevention.

## Background

Intimate partner violence (IPV) against women is a global human rights and public health concern. The WHO Multi-Country Study on Women's Health and Domestic Violence documented the widespread nature of IPV [1], with lifetime prevalence of physical and/or sexual partner violence among ever-partnered women in the fifteen sites surveyed ranging from 15% in Ethiopia province to 71% in Japan. Between 4%-54% of respondents reported experiencing this violence in the year prior to the survey. In addition to being a concern in its own right, IPV is associated with a range of adverse physical, mental, sexual and reproductive health outcomes [2-8].

Designing effective IPV prevention programmes involves identification of risk factors--both those that are direct causes of IPV, and those that point to common characteristics of victims and/or perpetrators thus allowing appropriate tailoring and targeting of services. Studies in various countries have identified a range of factors that influence IPV risk [9-13], but in some cases, protective factors in one setting may be ineffective or actually increase risk in another [14]. For the purposes of intervention development, there is considerable interest in identifying a set of risk and protective factors for IPV that behave consistently across settings, to maximise chances of intervention success and minimise chances of inadvertently doing harm.

It is difficult to make comparisons between settings using existing individual studies as differences in identified risk factors may either be methodological artefacts or a real reflection of contrasting phenomena. Selected Demographic and Health Surveys [12,15] have added a Domestic Violence Module; however, country-level adaptations to the module and interviewer training procedures still limit their comparability. Standardisation is very important in a research field where even individual interviewer effects have a profound effect on level of disclosure[16].

**Figure 1:**
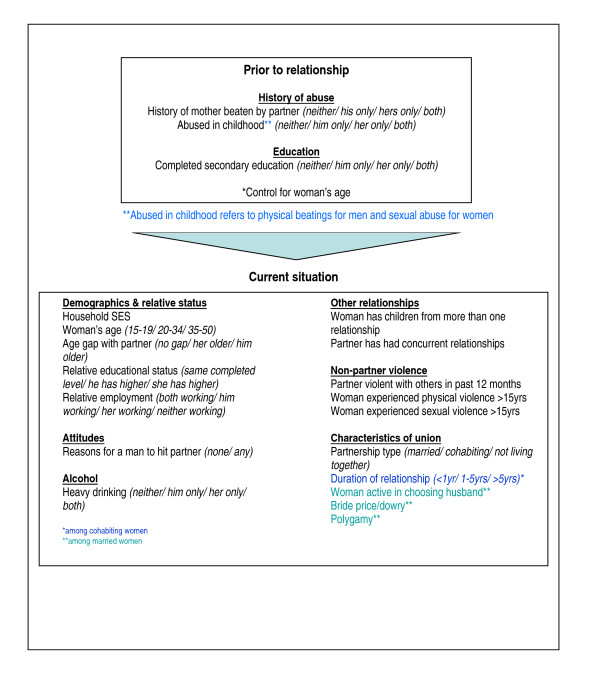
**Predictors of current IPV - the 'relationship approach'**.

We use population-based data from the WHO Multi-Country Study on Women's Health and Domestic Violence, which was specifically designed to better understand the factors associated with violence in different settings. Comparability of data was maximised through use of a standardised questionnaire, standardised interviewer training and data-collection procedures across all participating sites, and a rigorous set of quality control procedures. We drew on current models of IPV risk, including those of Heise [17,18] and Jewkes [19] to develop a 'relationship' approach to assessing IPV risk. The characteristics and experiences of both the victim and the perpetrator are considered - in terms of what happened to each before they entered into the relationship, and their relative situations within the relationship - alongside features and dynamics of the relationship itself (See Figure 1). Our goal is to identify factors that appear to consistently increase or decrease risk of partner violence across settings, and to identify where there are differences in patterns of association between sites.

## Methods

Details of the study methods, sampling, response rates, and prevalence of different types of partner violence in each setting have been reported elsewhere [1] (see Additional file 1). Briefly, population-based surveys were conducted in Bangladesh, Brazil, Ethiopia, Japan, Namibia, Peru, Republic of Tanzania, Samoa, Serbia and Montenegro, and Thailand. In five countries surveys were done in the capital or another large city and one predominantly rural province. In the other five countries, only one site was surveyed because of logistical and financial considerations.

Trained female interviewers completed interviews with one randomly selected woman aged 15-49 from each sampled household. 24,097 women were interviewed in total, using a standardised questionnaire which was developed by the study team and translated into 14 languages.

Specially developed ethical guidelines emphasised the importance of ensuring confidentiality and privacy, both to protect the safety of respondents and field staff and to improve the quality of the data [20]. Ethical approval for the study was obtained from WHO's ethical review group (WHO Secretariat Committee for Research in Human Subjects), from the local institutions and, where necessary, national ethical review boards.

## Measures and Data Analysis

### IPV Outcome

**Figure 2:**
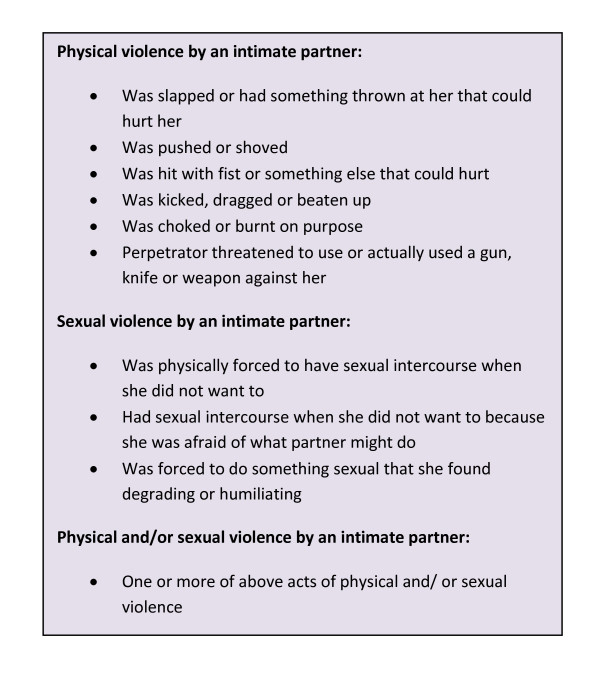
**Operational definitions of intimate partner violence**.

Currently- or previously-partnered women were asked a series of questions about whether they had ever experienced specific violent acts (see Figure 2), and if so whether this had happened in the 12 months preceding the survey.^1 ^The analysis compared women who reported having experienced any act of physical and/or sexual violence in the past year (conceptualised as 'current violence') with women who did not report any partner violence ('current' versus 'never'). Those who had experienced partner violence during their lifetime but not in the past year were excluded from the analysis so as not to dilute associations.

### Risk-factors for IPV

Variables are outlined in Figure 1, and consist of the woman's past history, her current/most recent partner's past history, the current situation of each of them (including relative status measures) and characteristics of the relationship and household. Variables were conceptualised as 'prior to relationship' if they preceded the relationship or 'current situation' if they related to the current situation within the relationship.

### Analysis Strategy

All analyses were conducted separately by study site, using STATA 10.0. We used bivariate logistic regression to estimate the crude associations between each exposure variable and IPV, and to select variables for the multivariate analysis. Multivariate logistic regression was then used to model factors associated with past year IPV, separately for 'prior to relationship' characteristics, and 'current situation' variables. This chronological separation allowed us to explore the effects of the early-life exposures independently of the later-life variables, which may be on the causal pathway (and thus attenuate the associations) between early-life experiences and later IPV. Clustering of outcomes in each site was 'small' (all intra-class correlation coefficients were less than 0.06) [21]; hence we present results unadjusted for clustering.

We included the same variables (decided upon *a priori*) across the site-models except for the addition of certain relationship characteristics (polygamy and bride price/dowry) that were only relevant in some sites. As our overall aim was to identify similarities and differences in patterns of association between settings, we did not attempt to fit the most parsimonious model for each site. Neither did we place too much emphasis on the statistical significance of individual associations. Instead we focused on exploring the extent to which, keeping all other features of the model constant, patterns of associations were similar or different between sites.

When reporting results, we consider odds ratios (OR) between 0.95 > OR < 1.05 as indicative of no association, ORs of 1.05 or greater as risk-factors for IPV, and ORs of 0.95 or less as protective-factors for IPV. We use the terms risk- and protective-factors loosely to indicate the direction of association with IPV rather than to imply causality, as we are analysing cross-sectional data. Statistical significance is considered at the 5% level.

## Results

19,517 women reported having ever had a partner and were thus asked about partner violence. In total, having excluded women reporting lifetime but not past-year experience of violence, and those with missing data for key variables in the models, 15,207 women were included in the 'prior to relationship' analyses, and 15,058 in the 'current situation' analyses (see Tables 1 and 2).

**Table 1:** Descriptive data for 'prior to relationship' variables, and AORs* and 95%CIs for associations with current IPV among ever partnered women

	BGD rural (n = 934)		BGD urban (n = 1053)		BRA rural (n = 925)		BRA urban (n = 756)		ETH rural (n = 1873)		JPN urban (n = 1141)		NAM urban (n = 1162)			
	%	**AOR (95%CI)**	**%**	**AOR (95%CI)**	**%**	**AOR (95%CI)**	**%**	**AOR (95%CI)**	**%**	**AOR (95%CI)**	**%**	**AOR (95%CI)**	**%**	**AOR (95%CI)**		

**Education**																

Secondary completion**																

Neither completed	38	-	11	-	64	-	41	-	70	-	1	-	36	-		

He completed	22	0.75 (0.52 - 1.1)	13	0.45 (0.26 - 0.78)	5	0.76 (0.33 - 1.7)	10	0.37 (0.15 - 0.92)	7	1.1 (0.73 - 1.6)	2	--	16	0.74 (0.49 - 1.1)		

She completed	7	0.88 (0.50 - 1.6)	4	1.0 (0.47 - 2.3)	13	1.0 (0.60 - 1.7)	11	0.39 (0.16 - 0.91)	2	1.6 (0.66 - 3.8)	6	--	7	0.50 (0.27 - 0.90)		

Both completed	34	0.57 (0.41 - 0.80)	71	0.28 (0.18 - 0.43)	9	0.38 (0.16 - 0.91)	32	0.40 (0.22 - 0.74)	2	0.44 (0.21 - 0.92)	91	--	28	0.33 (0.22 - 0.49)		

Missing data	0	--	0	--	10	1.55 (0.91 - 2.7)	5	0.51 (0.15 - 1.8)	20	1.1 (0.88 - 1.5)	0	--	13	0.70 (0.44 - 1.1)		

**History of abuse**																

Reported history of mother abused																

Neither mother	81	-	79	-	69	-	69	-	74	-	80	-	76	-		

His mother	10	3.2 (1.9 - 5.2)	7	4.4 (2.5 - 7.8)	11	2.8 (1.7 - 4.5)	12	1.9 (0.95 - 3.8)	2	2.4 (1.0 - 5.8)	5	1.6 (0.52 - 4.7)	3	4.7 (2.2 - 9.8)		

Her mother	7	2.7 (1.6 - 4.7)	11	3.4 (2.2 - 5.3)	15	1.6 (1.0 - 2.6)	15	2.3 (1.3 - 4.2)	20	2.4 (1.9 - 3.2)	13	1.8 (0.84 - 3.8)	19	3.0 (2.1 - 4.1)		

Both	2	13.2 (3.0 - 58.5)	3	3.9 (1.8 - 8.4)	4	4.1 (2.0 - 8.2)	5	3.7 (1.6 - 8.6)	4	2.5 (1.1 - 5.5)	2	0.47 (0.06 - 4.0)	2	2.2 (0.93 - 5.3)		

History of abuse as child																

Neither	90	-	83	-	78	-	74	-	96	-	88	-	90	-		

Him	9	2.4 (1.4 - 4.2)	11	1.6 (1.1 - 2.5)	17	1.9 (1.2 - 2.9)	20	1.7 (0.98 - 3.1)	4	2.9 (1.2 - 7.0)	4	6.6 (2.4 - 18.2)	6	1.8 (1.0 - 3.1)		

Her	1	2.5 (0.59 - 10.6)	5	2.5 (1.3 - 4.7)	3	4.5 (2.1 - 9.6)	5	2.9 (1.2 - 7.1)	0.1	--	8	5.3 (2.6 - 10.8)	4	1.6 (0.80 - 3.3)		

Both	0.3	1.6 (0.13 - 20.7)	1	15.2 (1.9 - 123.8)	2	3.4 (1.2 - 10.1)	2	5.8 (1.9 - 17.9)	0	--	1	15.1 (3.3 - 68.5)	0.4	0.69 (0.07 - 6.6)		

	PER rural (n = 1008)		PER urban (n = 746)		SMA (n = 932)		SRB urban (n = 957)		THA rural (n = 781)		THA urban (n = 848)		TZA rural (n = 922)		TZA urban (n = 1169)	

	%	AOR (95%CI)	%	AOR (95%CI)	%	AOR (95%CI)	%	AOR (95%CI)	%	AOR (95%CI)	%	AOR (95%CI)	%	AOR (95%CI)	%	AOR (95%CI)

**Education**																

Secondary completion**																

Neither completed	50	-	10	-	43	-	3	-	66	-	37	-	13	-	7	-

He completed	21	0.93 (0.66 - 1.3)	15	1.5 (0.76 - 2.8)	13	0.68 (0.42 - 1.1)	4	1.1 (0.26 - 4.8)	7	0.86 (0.46 - 1.6)	14	1.3 (0.80 - 2.1)	19	0.47 (0.28 - 0.78)	15	1.4 (0.68 - 2.7)

She completed	3	0.79 (0.34 - 1.8)	5	1.8 (0.74 - 4.4)	6	0.69 (0.34 - 1.4)	3	0.88 (0.18 - 4.4)	5	0.46 (0.20 - 1.1)	6	0.78 (0.37 - 1.6)	9	0.50 (0.27 - 0.93)	7	1.1 (0.50 - 2.5)

Both completed	23	0.51 (0.36 - 0.71)	68	0.65 (0.37 - 1.2)	10	0.59 (0.33 - 1.0)	87	0.22 (0.06 - 0.75)	13	0.63 (0.37 - 1.1)	34	0.96 (0.66 - 1.4)	57	0.32 (0.20 - 0.51)	64	1.4 (0.73 - 2.6)

Missing data	3	3.0 (1.3 - 6.9)	2	3.0 (0.90 - 9.7)	28	1.1 (0.78 - 1.6)	3	1.1 (0.22 - 5.9)	9	1.2 (0.68 - 2.1)	10	0.81 (0.43 - 1.5)	3	0.33 (0.13 - 0.83)	8	1.4 (0.68 - 3.1)

**History of abuse**																

Reported history of mother abused																

Neither mother	34	-	49	-	54	-	80	-	65	-	69	-	47	-	68	-

His mother	15	2.2 (1.4 - 3.3)	15	2.5 (1.4 - 4.2)	4	2.7 (1.4 - 5.5)	6	1.3 (0.42 - 4.3)	7	1.2 (0.63 - 2.3)	5	0.75 (0.34 - 1.6)	6	3.4 (1.8 - 6.3)	3	2.5 (1.2 - 5.1)

Her mother	28	1.7 (1.2 - 2.4)	23	2.3 (1.5 - 3.7)	36	1.8 (1.3 - 2.5)	11	1.6 (0.64 - 4.0)	23	1.7 (1.2 - 2.5)	23	1.4 (1.0 - 2.1)	34	1.8 (1.3 - 2.4)	25	2.0 (1.5 - 2.8)

Both	22	2.9 (2.0 - 4.2)	14	3.1 (1.8 - 5.2)	6	4.7 (2.6 - 8.5)	3	0.69 (0.12 - 4.0)	6	2.5 (1.3 - 4.8)	3	3.1 (1.3 - 7.6)	13	2.3 (1.5 - 3.6)	4	3.1 (1.7 - 5.8)

History of abuse as child																

Neither	61	-	58	-	91	-	93	-	85	-	86	-	89	-	91	-

Him	32	1.5 (1.1 - 2.1)	25	2.2 (1.4 - 3.3)	7	2.9 (1.7 - 4.9)	6	9.5 (3.9 - 23.4)	11	3.0 (1.9 - 4.9)	8	3.2 (1.9 - 5.6)	7	2.5 (1.4 - 4.3)	5	1.6 (0.89 - 2.8)

Her	4	2.4 (1.2 - 7.8)	9	2.2 (1.2 - 3.9)	1	1.4 (0.45 - 4.6)	1	2.8 (0.41 - 18.5)	3	3.8 (1.6 - 9.4)	5	1.9 (1.0 - 3.7)	4	1.7 (0.82 - 3.5)	3	0.72 (0.32 - 1.7)

Both	3	3.6 (1.5 - 8.6)	8	6.3 (3.3 - 12.2)	1	7.0 (0.62 - 80.6)	0.3	10.3 (0.72 - 147)	1	23.9 (2.9 - 200.1)	1	3.9 (0.82 - 18.3)	1	1.3 (0.19 - 9.2)	1	2.8 (0.63 - 12.1)

*Adjusted for all variables in Table and woman's age

**For Bangladesh Urban & Rural, Ethiopia Rural and Tanzania Urban & Rural, this variable refers to completed primary education

**Table 2:** Descriptive data for 'current situation' in relationship variables, and AORs* and 95%CIs for associations with current IPV among ever partnered women

	BGD rural (n = 926)		BGD urban (n = 1050)		BRA rural (n = 919)		BRA urban (n = 746)		ETH rural (n = 1861)		JPN urban (n = 1075)		NAM urban (n = 1154)			
	**%**	**AOR (95%CI)**	**%**	**AOR (95%CI)**	**%**	**AOR (95%CI)**	**%**	**AOR (95%CI)**	**%**	**AOR (95%CI)**	**%**	**AOR (95%CI)**	**%**	**AOR (95%CI)**		

**Demographics**																

SES																

Low	76	-	72	-	26	-	34	-	84	-	30	-	27	-		

Medium	20	0.71 (0.49 - 1.0)	22	0.68 (0.48 - 0.96)	64	0.63 (0.40 - 0.99)	40	0.99 (0.54 - 1.8)	14	0.91 (0.66 - 1.3)	23	0.71 (0.33 - 1.5)	26	1.3 (0.81 - 2.0)		

High	4	0.69 (0.34 - 1.4)	7	0.33 (0.17 - 0.66)	11	0.75 (0.35 - 1.6)	26	0.25 (0.09 - 0.67)	2	0.53 (0.25 - 1.1)	47	0.24 (0.10 - 0.58)	47	0.94 (0.61 - 1.5)		

Woman's age																

35+	35	-	28	-	39	-	46	-	41	-	54	-	36	-		

20-35 yrs	57	2.1 (1.5 - 3.1)	64	2.7 (1.8 - 4.0)	54	1.7 (1.0 - 2.7)	47	2.4 (1.2 - 4.6)	55	2.4 (1.9 - 3.1)	44	0.78 (0.38 - 1.62)	58	0.93 (0.62 - 1.4)		

15-19 yrs	8	3.2 (1.7 - 6.2)	9	4.1 (2.2 - 7.7)	8	2.9 (1.2 - 6.7)	6	4.2 (1.3 - 13.1)	4	1.7 (0.97 - 3.1)	2	1.1 (0.09 - 13.6)	6	1.1 (0.47 - 2.4)		

Age gap with partner (> = 5 yrs)																

None	11	-	16	-	52	-	58	-	11	-	75	-	55	-		

She is > = 5 yrs older	0	--	0.1	--	5	0.89 (0.33 - 2.4)	6	1.3 (0.42 - 3.8)	1	1.1 (0.31 - 3.6)	2	0.65 (0.07 - 6.0)	2	1.2 (0.46 - 3.2)		

He is > = 5 yrs older	89	0.90 (0.56 - 1.44)	84	0.81 (0.56 - 1.2)	43	0.99 (0.67 - 1.5)	36	0.89 (0.50 - 1.6)	88	1.3 (0.93 - 1.7)	24	0.92 (0.42 - 2.0)	43	0.96 (0.69 - 1.34)		

Relative education																

Same level	64	-	61	-	60	-	61	-	71	-	92	-	54	-		

He has higher	28	0.74 (0.50 - 1.09)	34	1.1 (0.84 - 1.6)	11	1.6 (0.83 - 2.9)	17	0.73 (0.35 - 1.6)	8	1.0 (0.50 - 2.1)	2	1.6 (0.33 - 8.3)	22	1.2 (0.80 - 1.8)		

She has higher	7	0.83 (0.58 - 1.19)	5	1.1 (0.74 - 1.7)	19	1.6 (0.97 - 2.7)	18	1.1 (0.53 - 2.1)	2	1.3 (0.82 - 1.9)	6	0.94 (0.25 - 3.6)	12	1.0 (0.63 - 1.7)		

Missing	-	--	-	--	10	1.5 (0.80 - 2.7)	5	0.1 (0.02 - 0.50)	20	1.4 (1.1 - 1.8)	-	--	13	0.98 (0.58 - 1.6)		

Relative employment																

Both working	18	-	15	-	31	-	56	-	36	-	53	-	48	-		

Man working	74	0.58 (0.40 - 0.83)	80	0.88 (0.60 - 1.3)	52	0.83 (0.52 - 1.3)	31	1.3 (0.70 - 2.4)	62	0.99 (0.79 - 1.2)	40	1.4 (0.7 - 3.0)	35	1.19 (0.82 - 1.7)		

Woman working	2	1.0 (0.40 - 2.6)	1	0.72 (0.10 - 5.2)	7	1.0 (0.48 - 2.2)	8	2.8 (1.2 - 6.7)	1	0.75 (0.26 - 2.1)	5	6.0 (1.5 - 23.3)	7	0.83 (0.43 - 1.6)		

Neither working	6	0.99 (0.52 - 1.9)	4	0.75 (0.33 - 1.7)	10	1.2 (0.63 - 2.4)	5	1.1 (0.36 - 3.3)	1	0.54 (0.20 - 1.5)	2	No cases	10	1.9 (1.1 - 3.2)		

**Attitudes**																

Reasons for a man to hit partner																

None	20	-	47	-	69	-	90	-	8	-	82	-	79	-		

Any	80	2.5 (1.7 - 3.6)	53	1.9 (1.4 - 2.5)	31	1.5 (1.0 - 2.2)	10	2.8 (1.4 - 5.8)	92	1.6 (1.1 - 2.2)	18	1.7 (0.82 - 3.4)	21	1.4 (0.99 - 2.1)		

**Alcohol use**																

Heavy drinking																

Neither	100	-	99	-	72	-	86	-	85	-	65	-	62	-		

He	0	--	1	6.5 (1.4 - 30.9)	24	3.1 (2.1 - 4.6)	11	4.3 (2.2 - 8.4)	13	2.1 (1.5 - 3.0)	33	1.8 (0.94 - 3.6)	28	1.8 (1.2 - 2.5)		

She	0	--	0	--	2	5.1 (1.7 - 15.5)	2	8.1 (2.3 - 28.9)	1	1.3 (0.41 - 4.4)	1	1.6 (0.21 - 12.5)	4	2.3 (1.1 - 4.5)		

Both	0	--	0	--	2	8.6 (2.8 - 26.7)	1	13.9 (1.9 - 101.0)	1	2.8 (0.79 - 9.9)	1	6.3 (1.0 - 38.0)	6	5.4 (2.9 - 9.9)		

**Non-intimate-partner violence**																

Victim of sexual abuse (>15 yrs)																

No	99.6	-	94	-	96	-	94	-	99.7	-	97	-	95	-		

Yes	0.4	--	6	1.2 (0.67 - 2.2)	4	2.2 (0.92 - 5.2)	6	2.3 (0.97 - 5.6)	0.3	--	3	7.1 (2.2 - 22.5)	5	7.3 (3.7 - 14.1)		

Victim of physical abuse (>15 yrs)																

No	93	-	86	-	88	-	80	-	96	-	96	-	82	-		

Yes	7	1.6 (0.93 - 2.8)	14	1.3 (0.86 - 1.9)	12	1.8 (1.1 - 3.1)	20	2.1 (1.1 - 3.9)	4	2.6 (1.4 - 5.0)	4	5.4 (2.0 - 14.4)	18	2.4 (1.6 - 3.4)		

Partner has fought with other man (past yr)																

No	96	-	96	-	92	-	91	-	89	-	97	-	90	-		

Yes	4	5.7 (2.3 - 14.3)	4	4.5 (1.9 - 10.5)	8	4.1 (2.3 - 7.2)	9	3.2 (1.6 - 6.7)	11	4.2 (2.7 - 6.7)	3	11.3 (3.7 - 34.3)	10	2.5 (1.5 - 4.0)		

**Other relationships**																

Woman has children from >1 father																

1 father (>1 kid)	77	-	63	-	53	-	43	-	78	-	71	-	28	-		

>1 father	1	2.2 (0.66 - 7.4)	1	4.9 (0.76 - 31.2)	18	1.1 (0.61 - 1.8)	11	2.2 (0.88 - 5.5)	10	0.99 (0.68 - 1.4))	1	--	30	1.4 (0.91 - 2.3)		

0-1 kids	21	0.59 (0.38 - 0.94)	36	0.92 (0.64 - 1.3)	30	0.85 (0.48 - 1.5)	46	0.98 (0.46 - 2.1)	12	0.98 (0.63 - 1.5)	28	--	42	1.4 (0.85 - 2.3)		

Partner has had concurrent relationship(s)																

No	95	-	94	-	69	-	81	-	73	-	91	-	57	-		

Yes	3	5.8 (1.6 - 21.7)	3	7.5 (2.4 - 23.1)	25	2.1 (1.4 - 3.1)	13	4.5 (2.3 - 8.8)	27	1.2 (0.97 - 1.6)	3	5.8 (1.7 - 19.8)	23	1.7 (1.2 - 2.4)		

DK/maybe	3	3.4 (1.3 - 9.2)	3	2.1 (0.85 - 5.1)	6	1.2 (0.52 - 2.6)	6	2.7 (1.0 - 7.0)	0.3	0.52 (0.10 - 2.8)	6	0.63 (0.15 - 2.6)	19	0.97 (0.6 - 1.5)		

**Characteristics of union**																

Any marriage ceremony																

Married	50	-	78	-	48	-	56	-	98	-	87	-	36	-		

No, living together	50	0.88 (0.66 - 1.2)	22	0.81 (0.57 - 1.1)	48	1.4 (0.89 - 2.1)	30	1.1 (0.57 - 2.1)	2	0.61 (0.30 - 1.3)	0.1	--	29	1.6 (1.1 - 2.5)		

Not living together	-	--	-	-	5	0.94 (0.34 - 2.6)	15	0.60 (0.20 - 1.8)	0.2	--	13	0.39 (0.08 - 1.8)	35	0.97 (0.62 - 1.5)		

	PER rural (n = 1005)		PER urban (n = 736)		SMA (n = 931)		SRB urban (n = 954)		THA rural (n = 772)		THA urban (n = 845)		TZA rural (n = 918)		TZA urban (n = 1166)	

	%	AOR (95%CI)	%	AOR (95%CI)	%	AOR (95%CI)	%	AOR (95%CI)	%	AOR (95%CI)	%	AOR (95%CI)	%	AOR (95%CI)	%	AOR (95%CI)

**Demographics**																

SES																

Low	47	-	11	-	15	-	18	-	9	-	10	-	86	-	65	-

Medium	34	1.4 (0.97 - 2.0)	22	0.57 (0.29 - 1.1)	50	0.54 (0.35 - 0.84)	44	0.66 (0.25 - 1.74)	49	0.67 (0.37 - 1.2)	25	0.84 (0.45 - 1.6)	10	0.72 (0.42 - 1.2)	23	1.1 (0.76 - 1.6)

High	19	0.61 (0.36 - 1.0)	66	0.56 (0.30 - 1.0)	36	0.34 (0.21 - 0.56)	38	0.41 (0.15 - 1.18)	42	0.45 (0.24 - 0.85)	65	0.74 (0.41 - 1.3)	4	1.1 (0.51 - 2.5)	12	1.3 (0.81 - 2.2)

Woman's age																

35+	42	-	42	-	44	-	53	-	59	-	48	-	28	-	31	-

20-35 yrs	54	1.4 (0.94 - 2.0)	52	4.6 (2.6 - 8.0)	53	1.6 (1.1 - 2.4)	44	2.04 (0.82 - 5.1)	38	1.1 (0.74 - 1.6)	49	1.8 (1.2 - 2.6)	63	1.9 (1.2 - 2.9)	60	2.0 (1.3 - 3.0)

15-19 yrs	4	2.4 (1.1 - 5.3)	5	11.8 (4.4 - 31.3)	2	2.9 (1.0 - 8.4)	3	16.2 (2.5 - 104.2)	3	1.7 (0.58 - 5.0)	3	3.1 (1.1 - 8.4)	9	3.4 (1.7 - 6.8)	9	2.0 (0.99 - 3.9)

Age gap with partner (> = 5 yrs)																

None	61	-	58	-	54	-	66	-	64	-	59	-	38	-	33	-

She is > = 5 yrs older	2	0.84 (0.26 - 2.8)	4	1.8 (0.67 - 5.0)	4	1.9 (0.84 - 4.1)	1	13.3 (1.5 - 115.5)	3	1.2 (0.46 - 3.1)	3	0.47 (0.14 - 1.6)	0.3	--	0.1	--

He is > = 5 yrs older	37	1.2 (0.87 - 1.7)	39	0.74 (0.48 - 1.1)	42	0.88 (0.64 - 1.2)	33	2.0 (0.91 - 4.4)	33	0.87 (0.59 - 1.3)	38	1.0 (0.69 - 1.4)	61	0.75 (0.55 - 1.0)	67	0.82 (0.60 - 1.1)

Relative education																

Same level	54	-	75	-	49	-	90	-	62	-	61	-	65	-	60	-

He has higher	38	1.0 (0.87 - 1.7)	18	1.4 (0.84 - 2.5)	14	0.92 (0.58 - 1.5)	4	4.6 (1.4 - 15.5)	18	1.3 (0.75 - 2.1)	20	1.4 (0.92 - 2.2)	23	1.5 (0.94 - 2.4)	25	0.77 (0.50 - 1.2)

She has higher	5	1.2 (0.81 - 1.9)	5	1.7 (0.82 - 3.4)	8	0.86 (0.45 - 1.7)	3	6.5 (1.5 - 27.1)	11	1.0 (0.62 - 1.7)	10	1.1 (0.62 - 1.9)	9	1.1 (0.74 - 1.6)	7	0.73 (0.48 - 1.1)

Missing	3	3.4 (1.3 - 9.3)	2	6.3 (1.8 - 22.7)	28	1.1 (0.74 - 1.6)	3	4.0 (1.0 - 15.3)	9	1.5 (0.80 - 2.8)	10	0.72 (0.37 - 1.4)	3	1.1 (0.43 - 2.9)	8	1.0 (0.55 - 2.0)

Relative employment																

Both working	51	-	59	-	43	-	-	--	75	-	70	-	61	-	40	-

Man working	37	1.1 (0.79 - 1.5)	26	0.79 (0.49 - 1.3)	43	0.89 (0.63 - 1.3)	-	--	17	0.88 (0.55 - 1.4)	23	0.65 (0.41 - 1.0)	33	1.1 (0.80 - 1.5)	46	0.91 (0.66 - 1.2)

Woman working	8	1.1 (0.63 - 2.0)	11	1.8 (0.99 - 3.4)	7	0.71 (0.35 - 1.4)	-	--	6	0.83 (0.39 - 1.8)	5	1.5 (0.74 - 3.2)	3	1.4 (0.55 - 3.6)	6	1.0 (0.55 - 2.0)

Neither working	3	2.2 (0.89 - 5.3)	4	1.2 (0.38 - 3.5)	7	1.2 (0.62 - 2.2)	-	--	3	2.0 (0.72 - 5.7)	2	6.4 (1.9 - 21.5)	3	0.92 (0.38 - 2.2)	8	0.72 (0.39 - 1.3)

**Attitudes**																

Reasons for a man to hit partner																

None	19	-	67	-	28	-	95	-	29	-	52	-	33	-	37	-

Any	81	1.5 (0.94 - 2.3)	33	1.4 (0.92 - 2.1)	72	1.5 (1.0 - 2.1)	5	1.3 (0.32 - 5.0)	71	0.95 (0.64 - 1.4)	48	0.95 (0.67 - 1.3)	67	1.8 (1.3 - 2.5)	63	1.9 (1.3 - 2.6)

**Alcohol use**																

Heavy drinking																

Neither	51	-	70	-	67	-	94	-	64	-	73	-	78	-	78	-

He	31	3.3 (2.3 - 4.6)	19	2.5 (1.5 - 4.1)	32	1.5 (1.1 - 2.1)	6	8.6 (3.5 - 21.3)	26	2.7 (1.9 - 4.0)	21	2.1 (1.4 - 3.1)	18	3.4 (2.3 - 5.1)	19	2.5 (1.8 - 3.6)

She	7	2.1 (1.1 - 3.7)	8	1.0 (0.48 - 2.1)	0.3	--	0.2	--	5	2.6 (1.3 - 5.4)	3	1.1 (0.40 - 2.9)	2	1.3 (0.44 - 4.1)	2	1.6 (0.56 - 4.8)

Both	12	11.8 (6.1 - 22.8)	4	1.1 (0.42 - 3.0)	0.3	--	0	--	5	6.0 (2.8 - 13.0)	3	2.6 (0.98 - 6.6)	2	4.3 (1.6 - 11.6)	1	2.9 (0.97 - 8.7)

**Non-intimate-partner violence**																

Victim of sexual abuse (>15 yrs)																

No	89	-	89	-	91	-	98	-	98	-	94	-	92	-	91	-

Yes	11	2.6 (1.5 - 4.5)	11	2.1 (1.2 - 3.9)	9	1.2 (0.69 - 2.0)	2	0.26 (0.03 - 2.6)	2	1.3 (0.41 - 4.3)	6	2.6 (1.3 - 5.3)	8	1.4 (0.78 - 2.4)	9	2.8 (1.7- 4.5)

Victim of physical abuse (>15 yrs)																

No	68	-	72	-	41	-	92	-	91	-	93	-	86	-	83	-

Yes	32	2.0 (1.4 - 2.7)	28	2.3 (1.5 - 3.5)	59	1.2 (0.85 - 1.6)	8	3.1 (1.1 - 8.6)	9	1.9 (1.1 - 3.4)	7	3.0 (1.6 - 5.5)	14	1.3 (0.82 - 1.9)	17	0.93 (0.63 - 1.4)

Partner has fought with other man (past yr)																

No	78	-	91	-	82	-	88	-	86	-	88	-	95	-	95	-

Yes	22	2.3 (1.5 - 3.6)	9	2.5 (1.3 - 4.7)	18	3.7 (2.5 - 5.4)	12	4.6 (2.0 - 10.6)	14	2.6 (1.6 - 4.1)	12	3.6 (2.2 - 5.8)	5	1.9 (0.84 - 4.2)	5	1.7 (0.89 - 3.2)

**Other relationships**																

Woman has children from >1 father																

1 father (>1 kid)	69	-	49	-	70	-	42	-	55	-	47	-	61	-	45	-

>1 father	8	2.2 (1.1 - 4.8)	9	2.2 (0.99 - 5.1)	11	2.1 (1.2 - 3.6)	2	3.6 (0.30 - 41.6)	7	0.89 (0.41 - 1.9)	5	1.5 (0.64 - 3.3)	14	1.5 (0.91 - 2.4)	16	1.4 (0.90 - 2.2)

0-1 kids	23	0.89 (0.55 - 1.4)	41	0.83 (0.47 - 1.5)	19	1.6 (0.94 - 2.6)	56	1.5 (0.53 - 4.2)	38	1.4 (0.89 - 2.1)	48	0.96 (0.62 - 1.5)	25	0.81 (0.51 - 1.3)	40	0.65 (0.42 - 1.0)

Partner has had concurrent relationship(s)																

No	67	-	69	-	81	-	87	-	72	-	66	-	61	-	53	-

Yes	20	4.0 (2.6 - 6.2)	17	5.2 (3.1 - 8.7)	17	2.0 (1.3 - 3.0)	4	6.6 (2.0 - 21.7)	19	2.4 (1.6 - 3.7)	17	2.1 (1.4 - 3.3)	21	3.1 (2.1 - 4.5)	16	2.9 (1.9 - 4.2)

DK/maybe	13	2.4 (1.5 - 3.9)	13	2.7 (1.5 - 4.6)	2	--	8	2.3 (0.75 - 6.8)	9	3.4 (1.9 - 6.0)	16	1.4 (0.86 - 2.2)	18	2.1 (1.5 - 3.2)	31	1.3 (0.91 - 1.8)

**Characteristics of union**																

Any marriage ceremony																

Married	59	-	53	-	74	-	69	-	89	-	82	-	57	-	61	-

No, living together	36	1.2 (0.81 - 1.7)	27	1.1 (0.65 - 1.7)	26	2.5 (0.93 - 6.5)	11	3.1 (1.1 - 8.6)	10	1.6 (0.90 - 2.7)	17	0.84 (0.53 - 1.3)	37	1.2 (0.84 - 1.6)	24	1.8 (1.3 - 2.6)

Not living together	6	0.75 (0.34 - 1.7)	20	0.52 (0.26 - 1.0)	0	--	20	0.34 (0.07 - 1.7)	1	--	1	--	6	0.39 (0.17 - 0.88)	15	0.67 (0.40 - 1.1)

*Adjusted for all variables in Table, and parity

Percentage distributions and adjusted odds ratios for all variables in the multivariate models are presented in Table 1 (prior to relationship), and Tables 2 and 3 (current relationship).

**Table 3:** Descriptive data for additional 'characteristics of union' variables, and AORs* and 95%CIs for associations with current IPV among cohabiting and married women

	BGD rural		BGD urban		BRA rural		BRA urban		ETH rural		JPN urban		NAM urban			
	**%**	**AOR (95%CI)**	**%**	**AOR (95%CI)**	**%**	**AOR (95%CI)**	**%**	**AOR (95%CI)**	**%**	**AOR (95%CI)**	**%**	**AOR (95%CI)**	**%**	**AOR (95%CI)**		

*Among cohabiting*	(n = 926)		(n = 1050)		(n = 870)		(n = 639)		(n = 1859)		(n = 833)		(n = 754)			

Duration of relationship																

> = 5 yrs	80	-	75	-	68	-	68	-	85	-	-	-	61	-		

1-5 yrs	16	0.63 (0.36 - 1.1)	21	0.94 (0.61 - 1.4)	24	0.68 (0.38 - 1.2)	26	0.67 (0.30 - 1.5)	11	0.68 (0.45 - 1.0)	-	--	30	0.83 (0.50 - 1.4)		

<1 yr	4	0.57 (0.21 - 1.5)	3	0.44 (0.17 - 1.1)	7	1.3 (0.60 - 3.0)	6	0.86 (0.23 - 3.3)	3	0.49 (0.24 - 0.97)	-	--	9	0.95 (0.43 - 2.1)		

*Among cohabiting*	(n = 926)		(n = 1046)		(n = 870)		(n = 639)		(n = 1821)		(n = 830)		(n = 407)			

Choice of husband																

She took part	5	-	16	-	98	-	98	-	6	-	98	-	72	-		

He/family chose	95	0.66 (0.34 - 1.3)	84	0.63 (0.43 - 0.93)	2	1.0 (0.17 - 6.4)	2	6.3 (1.3 - 31.7)	94	1.6 (1.0 - 2.4)	2	3.2 (0.51 - 19.6)	28	0.62 (0.28 - 1.4)		

Bride price/dowry		(binary)		(binary)												

Dowry	54	1.8 (1.3 - 2.4)	12	2.3 (1.5 - 3.5)					10	1.4 (1.0 - 2.1)			9	1.7 (0.57 - 5.0)		

Bride price	1	--	1	--					7	0.65 (0.45 - 0.95)			19	0.78 (0.32 - 1.9)		

None	45	-	87	-					80	-			72	-		

4	-		-						2	0.57 (0.30 - 1.1)						

Polygamy																

No	98	-	97	-					69	-			76	-		

Yes	2	1.66 (0.58 - 4.7)	3	3.0 (1.2 - 7.1)					31	1.3 (0.83 - 2.0)			11	2.6 (1.0 - 6.6)		

Don't know	0.3	--	-	--					0.1	1.1 (0.12 - 10.2)			13	--		

	PER rural		PER urban		SMA		SRB urban		THA rural		THA urban		TZA rural		TZA urban	

	%	AOR (95%CI)	%	AOR (95%CI)	%	AOR (95%CI)	%	AOR (95%CI)	%	AOR (95%CI)	%	AOR (95%CI)	%	AOR (95%CI)	%	AOR (95%CI)

*Among cohabiting*	(n = 946)		(n = 595)		(n = 931)		(n = 755)		(n = 769)		(n = 836)		(n = 870)		(n = 995)	

Duration of relationship																

> = 5 yrs	76	-	69	-	68	-	74	-	83	-	74	-	64	-	63	-

1-5 yrs	20	1.1 (0.64 - 1.8)	23	1.8 (0.91 - 3.5)	24	1.3 (0.79 - 2.0)	20	1.3 (0.41 - 4.0)	13	1.3 (0.71 - 2.4)	20	1.4 (0.88 - 2.3)	30	0.94 (0.60 - 1.5)	29	0.76 (0.50 - 1.2)

<1 yr	4	1.0 (0.39 - 2.8)	7	2.8 (1.0 - 7.6)	8	1.6 (0.76 - 3.3)	6	1.9 (0.29 - 12.6)	4	0.72 (0.25 - 2.0)	5	1.3 (0.58 - 2.9)	6	0.45 (0.20 - 1.0)	8	0.61 (0.30 - 1.2)

*Among cohabiting*	(n = 587)		(n = 383)		(n = 689)		(n = 650)		(n = 690)		(n = 681)		(n = 529)		(n = 709)	

Choice of husband																

She took part	80	-	87	-	96	-	99	-	85	-	91	-	92	-	87	-

He/family chose	20	0.82 (0.47 - 1.4)	13	0.37 (0.12 - 1.1)	4	0.37 (0.12 - 1.15)	1	135.7 (6.4 - 2872.5)	15	0.93 (0.54 - 1.6)	9	0.59 (0.26 - 1.3)	8	1.3 (0.61 - 2.4)	13	1.3 (0.69 - 2.2)

Bride price/dowry										(binary)		(binary)				

Dowry									0.4		1		1	0.47 (0.04 - 6.24)	7	1.0 (0.30 - 3.5)

Bride price									93	0.49 (0.25 - 0.98)	84	0.85 (0.49 - 1.5)	94	1.34 (0.53 - 3.43)	87	1.6 (0.67 - 4.0)

None									7	-	16	-	6	-	5	-

4																

Polygamy																

No													81	-	82	-

Yes													18	2.4 (1.4 - 4.1)	13	1.1 (0.67 - 1.9)

Don't know													1		5	----

*Adjusted for all variables in same and above sections of this Table, and for all variables in Table 2

### Prior to relationship

#### Education

Bivariate analysis of educational level indicated a reduction in IPV risk associated with secondary education for both the woman and her partner, but showed less consistent evidence of a protective effect of primary education. Therefore, when considering the woman and her partners' education in combination, we focused on complete versus incomplete secondary education (except for Bangladesh, Ethiopia and Tanzania where we examined primary completion because of extremely low secondary school enrolment). Achieving secondary education (or primary for Bangladesh, Ethiopia and Tanzania) by either the woman or her partner was associated with decreased IPV in almost two thirds of the sites (3 significant for each partner), when compared to situations where neither the woman nor her partner completed the level. However, the most consistent protective effect against IPV was observed where both the woman and her partner had completed the relevant schooling level (decreased risk in 12/14 sites, 10 significant). This most highly educated exposure group also had the lowest ORs for IPV in 10 out of 14 sites, compared to couples where one or both had not completed the level.

#### History of abuse

A history of abuse was strongly associated with the occurrence of IPV, with reports of abuse of the woman's mother, her partners' mother, or both (compared to no known reported abuse of either mother) being associated with increased risk of IPV in all sites (10 sites significant for women, 10 for partners, 12 for both). ORs for IPV tended to be highest where women reported that both their mothers and their partners' mothers experienced abuse (observed in 10/15 sites). Evidence from bivariate analysis in most sites showed that women who did not know whether their partners had histories of abuse were also at increased risk of IPV compared to those who reported their partners did not have these experiences.

Other experiences of violence were also associated with past year IPV, with a history of childhood sexual abuse of the woman, childhood beatings of her partner, or both consistently associated with increased risk of IPV, compared to no reports of abuse by either partner (15/15 sites for partners, all significant; 13/15 for women and both, 10 significant). Women in relationships where both she and her partner were abused in childhood are at the highest risk of IPV (true in 11/14 sites), (see Table 1). These exposure categories often contained small numbers of women. When child sexual abuse is considered in isolation at the bivariate level, its association with IPV is significant in 14/15 sites. Since CSA has also been linked to other intervening variables in the model, such as low educational attainment[22], the fact that CSA remains highly significant in the final model confirms it's importance as a risk factor for IPV.

While small numbers in the extreme exposure categories for the abuse variables result in very wide confidence intervals for some of the ORs, the consistency of 'dose-response' patterns observed for all variables in this model provides compelling evidence of the combined importance of childhood experiences of both the woman and her partner in relation to IPV in later life.

### Current situation model

#### Demographics

Younger age of women was strongly associated with increased risk of past year IPV in all sites (significant in 12). A similar pattern was seen in bivariate analysis for partner's age but this variable was excluded from multivariate models due to its strong correlation with the woman's age. In contrast, associations between IPV and an age-gap of at least 5 years between the woman and her partner were weak in most settings and the direction of the effect was context dependent. Older age of the woman was often associated with increased risk of IPV, but in only three out of fifteen sites was older age of the partner associated with increased risk of IPV. Weak associations were also seen in the other direction for age-gaps favouring either the woman or her partner.

There was some suggestion that inequality in educational level between a woman and her partner may increase her risk of experiencing IPV. This was true in nine out of 15 sites where the woman had the higher level of education (1 significant), and the same where her partner had the higher level. Associations tended to be weak, however, and some were also observed in the opposite direction.

There was no consistent pattern of association between IPV and relative employment status. Compared to couples in which both partners work, couples where just the man works appear to experience slightly lower levels of IPV in some settings (8/14, 2 sig). In some settings women who work when their partners do not may be at increased risk of IPV (6/14 sites, 2 significant). There is some evidence that women in relationships where neither she nor her partner work are at increased risk of IPV (8/13 sites, 2 significant). However, non-significant associations in opposite directions are also observed for these variables.

Higher socioeconomic status (SES) was associated with decreased IPV in fourteen sites (significant in 8 sites when comparing the highest status group to the lowest). This variable was more strongly associated with IPV before adjustment for other variables that may confound or mediate the effects of socioeconomic status on IPV risk.

#### Attitudes

In almost all sites, women who had attitudes supportive of a husband beating his wife had increased odds of IPV (13/15, 8 significant).

#### Alcohol

In all sites odds of IPV were higher in relationships where one or both partners had problems with alcohol, compared to relationships where neither of them did (him 14/14, 12 significant; her 10/11, 5 significant; both 11/11, 7 significant). In the majority of sites frequent drunkenness among men yielded higher ORs for IPV than problematic drinking by the woman (8/11), and in ten of the eleven sites ORs were higher when both had problems with alcohol.

#### Non-partner violence

Both a woman's experience of non-partner violence and her partner's involvement in fights with other men emerged as strong risk factors for IPV. Women's experiences of non-partner physical or sexual abuse over the age of 15 emerged as a risk-factor for IPV in almost all sites (14/15, 10 significant and 12/13, 6 significant respectively). Likewise, women whose partners were involved in a fight with another man in the past year experienced higher levels of IPV than those with partners who did not fight (significant in 13/15 sites). These factors were more strongly associated with IPV risk in the bivariate analysis. It is likely that both IPV and non-partner violence share common antecedents, such as CSA in the case of women, or a history of antisocial personality and alcohol abuse among men, which may account for all or part of this association [23,24]

#### Other relationships

Women with children from previous relationships were at increased risk of experiencing IPV in most sites (12/14, with 2 significant ORs). Women whose partners had had relationships with other women during their relationship also experienced higher levels of IPV than women with faithful partners (significant in 14/15 sites). Lack of knowledge/disclosure about a partner's involvement with other women was also associated with increased IPV in most sites.

#### Characteristics of the union

Women who were cohabiting with a partner without being formally married were at increased risk of IPV (10/12 sites, 3 significant). By contrast, women not living with their partners experienced lower levels of IPV (8/9 sites, 2 significant). There was some suggestion that those in newer relationships were at increased risk of IPV, with higher levels of IPV in relationships of less than five years compared to longer relationships, in half of the sites (mostly non-significant). There were also several sites where weak associations in the opposite direction were seen for the newest relationships (5).

A woman's participation in her choice of husband was associated with IPV differently across sites. In 6 out of 15 sites her lack of participation was associated with higher levels of IPV (3 significant), while in 8 sites it was associated with decreased IPV (1 significant).

Payment of dowry and bride price (compared to no marital exchange) was associated with IPV in some sites, though patterns of risk were difficult to interpret. In the 6 sites where dowry was paid, it was associated with higher levels of IPV in 4 sites (3 significant) and lower IPV in 1 site (not significant). Bride price was associated with decreased IPV in 4 sites (2 significant) and increased IPV in two sites (neither significant).

Women whose husbands had more than one wife were at increased risk of IPV in all 6 sites where polygamy is practised (3 statistically significant). The same was true for women who reported not knowing whether their husbands had other wives, compared to those who knew.

## Discussion

Despite the wide variations in the prevalence of IPV across the study sites, many risk factors appear to affect IPV risk similarly, with secondary education, high SES, and formal marriage offering protection, and alcohol abuse, cohabitation, young age, attitudes supporting wife beating, outside sexual relationships, experiencing childhood abuse, growing up with domestic violence, and perpetrating or experiencing other forms of violence in adulthood, increasing the risk of IPV. We also found that the strength of the association was greater when both the woman and her partner had the risk or protective factor, suggesting the possibility of achieving greater prevention impact through targeting programs to couples most at risk.

Overall, our analysis demonstrates far more consistency in risk and protective factors across sites than reported by Hindin and Kishor in their analysis of violence among couples from 10 recent Demographic and Health Surveys (DHS) [12]. Among the factors they examined, only alcohol consumption by the husband and exposure to inter-parental violence were consistently associated with a woman's risk of violence in her current relationship. The WHO study explored a wider range of potential risk and protective factors and was able to exert greater control over the training of interviewers and study implementation. Research has shown that disclosure of partner violence is highly influenced by interviewer factors as well as privacy and context of the interview--factors that are more difficult to control in national surveys designed for other purposes [16,25,26]. For this reason, underreporting and misclassification of abuse cases may have obscured some of the associations in the DHS analysis.

Our analysis confirms that completing secondary education has a protective effect on IPV risk, whereas primary education alone fails to confer similar benefits [27]. Studies in the USA and South Africa, for example, find an inverted U-shaped relationship between IPV and education, whereby protection from IPV is seen at the lowest and highest educational levels [28,29]. Results suggesting increased protection when both women and their partners complete secondary education, and those pointing towards increased IPV risk where there is disparity in educational attainment, confirm the importance of promoting equal access to education for boys and girls, as recommended by target 4 of the Gender Equality Goal of the Millennium Development Goals.

Higher socioeconomic status is generally associated with lower levels of physical and/or sexual partner violence. Even if it is not an independent or proximate risk factor but one that is partially confounded by or mediated through other factors (as suggested by the multivariate analysis), socioeconomic status of households should be taken into account when designing and targeting IPV intervention programmes [27].

Early life experiences of abuse (including the physical abuse of boys and the sexual abuse of girls) emerge as consistently strong risk factors for IPV. In order to intervene in this inter-generational cycle of abuse, interventions must address childhood abuse and respond appropriately to children who have witnessed IPV against their mothers. Although the importance of the sexual abuse of children and the witnessing of marital violence by children has been documented in other studies, the potential importance of the physical abuse of boys has received less attention and merits further exploration. The consistent association between IPV and other forms of violence against women also point to the need for integrated responses to violence across sectors and programmes [30,31]. For example, programming to support children exposed to marital violence, may help reduce their risk of violence in later life.

Male behaviours commonly associated with 'traditional' masculinity [32], such as having many sexual partners, controlling female behaviour, and fighting other men, are strongly associated with IPV across all sites. Women having children from another partnership, or, in some settings, working when her partner does not, also appear to increase her risk of IPV. These results highlight the need to engage with men and women to challenge norms around what is expected of, and deemed acceptable behaviour for both men and women. Promising research from Brazil, South Africa and Uganda highlights the potential impacts on partner violence, of programmes that tackle models of masculinity and address issues of gender norms [33].

Problematic alcohol use, among both women and their partners, is consistently and strongly associated with IPV. While it is difficult to establish the temporality of the observed associations, this relationship has been repeatedly been demonstrated in studies of IPV [12,34-36]. Health services, police and addiction programmes may therefore provide important entry points to identify and refer people who may be at risk of IPV. Interventions that try to address and change cultural norms supportive of excessive alcohol use might also be expected to have knock-on effects in terms of primary violence prevention [37].

Importantly, not all variables demonstrated consistent relationships with IPV across sites, suggesting that policymakers should be cautious about any 'one model fits all' approach to IPV prevention. For example, risk associated with age disparity among partners, a woman working where her partner does not, and a woman taking an active role in choosing her partner, varies by setting. What constitutes empowerment in one setting may represent an unacceptable transgression of gender norms elsewhere. Jewkes highlights that transgression of gender norms and failure to fulfil cultural expectations of good womanhood and successful manhood are among the most important triggers for intimate partner violence [19]. She argues that what constitutes a transgression may vary by setting, thus leading to cross-national variation in the behaviours that may emerge as risk factors. The fact that we sometimes but not invariably observe increased IPV risk associated with the higher relative status of a woman (for example if she works and her partner does not) can also be interpreted in the light of theories that risk of partner violence may increase during periods of transition in gender relations. Women who step into new roles before background gender norms have shifted may be at increased risk of violence [38]. It is thus important that prevention efforts engage with both men and women [19].

### Strengths and Limitations

The primary strength of our analysis is that it is based on fully comparable data from 15 culturally, economically and socially diverse sites. This type of comparison has not been possible to date in the field of IPV research, with the exception of the less tightly controlled DHS surveys. Obviously, the cross-sectional nature of this study limits the extent to which we can draw conclusions regarding temporality or the causal nature of observed associations. However, by distinguishing between early life and current characteristics, we do separate out those factors where temporality is clear from those where it is less certain.

A further limitation is that the study interviewed only women, and hence relies on women's reports of their partner's characteristics. The data on partner characteristics refers to the woman's current or most recent partner, who in some cases may not be the perpetrator of the reported violence. Since the analysis considers only past year IPV, however, the number of cases where the reported violence was perpetrated by a more distant partner is likely to be small. Any resulting misclassification would bias results towards the null rather than invalidate observed associations.

## Conclusions

The multi-faceted nature of the factors that influence partner violence highlights the need for a multi-sectoral response that combines development activities, including improved access to secondary education for girls and boys, with initiatives to transform gender norms and attitudes, address prior histories of abuse, and reduce harmful drinking. Since risk of IPV is highest in younger women, schools are also an important setting for primary prevention activities, with potential to address issues of relationships, gender roles, power and coercion within existing youth violence and bullying programmes. Although there is no magic bullet to reduce partner violence, the consistency of our findings across sites suggests that a prevention strategy, once validated and refined, might have relevance in a wide range of settings. Initiatives to reduce partner violence require commitment and vision--by the international community, local governments and civil society. The time to act is now. As highlighted in the recent UN Campaign against violence against women--Women Won't Wait--such responses are urgently needed.

## Abbreviations

IPV: intimate partner violence; CSA: childhood sexual abuse; DHS: Demographic and Health Survey; SES: socioeconomic status; WHO: World Health Organisation; UN: United Nations;

## Competing interests

The authors declare that they have no competing interests.

## Authors' contributions

CG-M, HAFMJ, ME, LH and CHW all participated in the study design and implementation. CG-M was the study coordinator. HAFMJ set up and supported data collection and processing in the countries and managed the central database. TA carried out the statistical analysis for this paper and drafted the manuscript. LH and CW helped draft the manuscript. KD and LK provided support with the statistical analysis and helped draft the manuscript. CG-M reviewed a draft of the manuscript. All authors read and approved the final manuscript.

## Note

^1^Women were defined as ever partnered if they had ever been married or lived with a partner (and therefore had been at risk of intimate partner violence). In practice, this definition varied slightly between countries in accordance with local notions of partnerships.

## Pre-publication history

The pre-publication history for this paper can be accessed here:

http://www.biomedcentral.com/1471-2458/11/109/prepub

## Supplementary Material

Additional file 1 **Prevalence of physical and/or sexual intimate partner violence among ever-partnered women, by site**. Prevalence data on lifetime and past-year experience of physical and/or sexual intimate partner violence among ever-partnered women for each of the sites included in the WHO study. These data are among those core study findings previously published in the Lancet (Garcia-Moreno C, Jansen HA, Ellsberg M, Heise L, Watts CH: **Prevalence of intimate partner violence: findings from the WHO multi-country study on women's health and domestic violence**. *Lancet *2006, **368**(9543):1260-1269. Click here for file
