# Supplementary material for: What factors are associated with recent intimate partner violence? findings from the WHO multi-country study on women's health and domestic violence
Source: BMC Public Health. 2011 Feb 16;11:109. doi: 10.1186/1471-2458-11-109 (PMC3049145; doi:10.1186/1471-2458-11-109)
Supplement: Additional file 1 — Prevalence of physical and/or sexual intimate partner violence among ever-partnered women, by site. Prevalence data on lifetime and past-year experience of physical and/or sexual intimate partner violence among ever-partnered women for each of the sites included in the WHO study. These data are among those core study findings previously published in the Lancet (Garcia-Moreno C, Jansen HA, Ellsberg M, Heise L, Watts CH: Prevalence of intimate partner violence: findings from the WHO multi-country study on women's health and domestic violence. Lancet 2006, 368(9543):1260-1269. [file 1471-2458-11-109-S1.DOC]

*Additional File 1: Prevalence of physical and/or sexual intimate partner violence among ever-partnered women, by site*

| Site | Total number of ever-partnered women | Ever  % (95%CI*) | Current  % (95%CI*) |
| --- | --- | --- | --- |
| Bangladesh city | 1373 | 53.4 (49.3 – 57.4) | 30.2 (26.5 – 33.9) |
| Bangladesh province | 1329 | 61.7 (58.6 – 64.8) | 31.9 (28.6 – 35.2) |
| Brazil city | 940 | 28.9 (25.5 – 32.4) | 9.3 (7.1 – 11.4) |
| Brazil province | 1188 | 36.9 (33.9 – 39.8) | 14.8 (12.9 – 16.7) |
| Ethiopia province | 2261 | 70.9 (69.0 – 72.7) | 53.7 (51.6 – 55.8) |
| Japan city | 1276 | 15.4 (13.4 – 17.4) | 3.8 (2.7 – 4.9) |
| Namibia city | 1367 | 35.9 (32.7 – 39.1) | 19.5 (16.7 – 21.9) |
| Peru city | 1086 | 51.2 (47.8 – 54.6) | 19.2 (16.7 – 21.6) |
| Peru province | 1534 | 69.0 (66.2 – 71.9) | 34.2 (31.5 – 36.9) |
| Samoa | 1204 | 46.1 (42.8 – 49.4) | 22.4 (19.9 – 24.9) |
| Serbia and Montenegro city | 1189 | 23.7 (20.7 – 26.7) | 3.7 (2.6 – 4.8) |
| Thailand city | 1048 | 41.1 (37.9 – 44.3) | 21.3 (18.7 – 23.8) |
| Thailand province | 1024 | 47.4 (43.6 – 51.1) | 22.9 (19.8 – 26.0) |
| United Republic of Tanzania city | 1442 | 41.3 (38.7 – 44.0) | 21.5 (19.0 – 23.6) |
| United Republic of Tanzania province | 1256 | 55.9 (52.3 – 59.4) | 29.1 (26.0 – 32.1) |

*Corrected for cluster sampling for all sites except Ethiopia where a simple random sample of women was used.
